# Spatial adjustment of bioenergetics, a possible determinant of contractile adaptation and development of contractile failure

**DOI:** 10.3389/fmmed.2023.1305960

**Published:** 2023-12-06

**Authors:** Marten Szibor, Marie Mühlon, Torsten Doenst, Jaakko L. O. Pohjoismäki

**Affiliations:** ^1^ Department of Cardiothoracic Surgery, Center for Sepsis Control and Care (CSCC), Jena University Hospital, Friedrich-Schiller University of Jena, Jena, Germany; ^2^ BioMediTech and Tampere University Hospital, Faculty of Medicine and Health Technology, Tampere University, Tampere, Finland; ^3^ Department of Environmental and Biological Sciences, University of Eastern Finland, Joensuu, Finland

**Keywords:** mitochondria, spatial bioenergetics, mammalian heart, contractility, heart failure

## Abstract

Cardiomyocytes depend on mitochondrial oxidative phosphorylation (OXPHOS) for energy metabolism, which is facilitated by the mitochondrial electron transfer system (ETS). In a series of thermogenic redox reactions, electrons are shuttled through the ETS to oxygen as the final electron acceptor. This electron transfer is coupled to proton translocation across the inner mitochondrial membrane, which itself is the main driving force for ATP production. Oxygen availability is thus a prerequisite for ATP production and consequently contractility. Notably, cardiomyocytes are exceptionally large cells and densely packed with contractile structures, which constrains intracellular oxygen distribution. Moreover, oxygen must pass through layers of actively respiring mitochondria to reach the ones located in the innermost contractile compartment. Indeed, uneven oxygen distribution was observed in cardiomyocytes, suggesting that local ATP supply may also vary according to oxygen availability. Here, we discuss how spatial adjustment of bioenergetics to intracellular oxygen fluctuations may underlie cardiac contractile adaptation and how this adaptation may pose a risk for the development of contractile failure.

## 1 Introduction

The adult human heart turns over the staggering amount of 6 kg of ATP per day to maintain contractility and organ homeostasis (Ingwall, 2002). The amount of ATP present in the organ, however, is comparatively low at about 0.7 g ATP per 250 g of cardiac tissue. This suffices to support organ function for about 10 heartbeats or, in terms of time, approximately 10 seconds (Ingwall, 2002). Continuous replenishment of ATP is thus a vital necessity.

**FIGURE 1:**
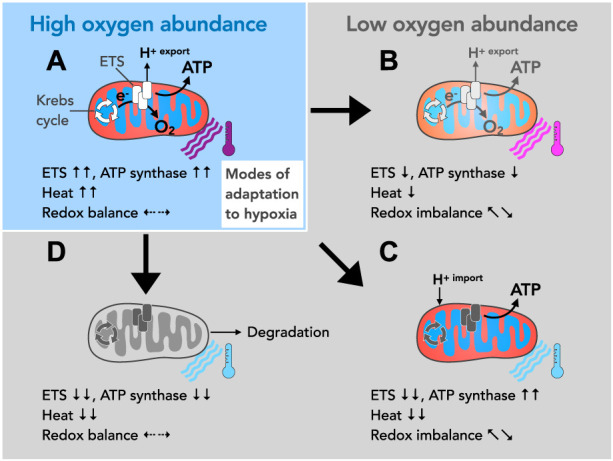
Possible modes of mitochondrial adaption to different oxygen levels. **(A)** Oxygen is the final electron acceptor for a series of thermogenic redox reactions facilitated by the ETS. These redox reactions are coupled to proton translocation (H^+^ export) across the inner mitochondrial membrane making the ETS the main building block for oxidative phosphorylation (OXPHOS), *i.e.*, mitochondrial ATP generation. ETS activity also keeps upstream metabolic circuits operational such as the tricarboxylic acid cycle (TCA, also known as the Krebs cycle), and produces heat and maintains cellular redox balance. **(B–D)** Due to their exceptional size, their contractile structures and their respiratory activity, radial oxygen gradients emerge within cardiomyocytes, which make adaptation mechanisms necessary. **(B)** Oxygen deprivation impairs ETS activity and thereby restricts proton translocation across the inner membrane. This decreases the driving force for ATP production and affects heat production and redox balance. More importantly, since well-oxygenated and less oxygenated mitochondrial populations may exist within one cardiomyocyte, differentially energized zones may build up and affect cellular functions. **(C)** According to the theory of proton conduction, hypoxic mitochondria may import the H^+^ gradient from well-oxygenated mitochondria. In principle, this mechanism would allow mitochondrial ATP production in the absence of oxygen. However, upstream metabolic cycles such as the TCA cycle, heat production and the redox balance would inevitably be disturbed. **(D)** The downregulation of ETS and ATP synthase activity makes the affected mitochondria susceptible to degradation (autophagy) but more importantly renders them metabolically quiescent. A partial downregulation of activity (as described in B) or a reversal in the mode of direction of the ATP synthase (as described in the text) may precede such state of metabolic hibernation.

The heart relies on the highly efficient oxidative phosphorylation (OXPHOS) system for adequate ATP production and enters contractile dysfunction when this system is faulty (Sabbah, 2020; Schwartz et al., 2022). The main building block of OXPHOS is the mitochondrial respiratory chain or electron transfer system (ETS), facilitating a series of thermogenic redox reactions for which oxygen serves as the terminal electron acceptor. Electron transfer through the ETS is coupled to proton translocation across the inner mitochondrial membrane, which in turn generates the main driving force for ATP production (Mitchell, 1961). Oxygen abundance is therefore a prerequisite for cardiac energy metabolism (bioenergetics) (Figure 1A). This way, OXPHOS provides the mammalian heart with an impressive reserve capacity (Cooke et al., 1998) without a need for anaerobic metabolism as seen in skeletal muscle (Hargreaves and Spriet, 2020).

Notably, across species cardiomyocytes are exceptionally large cells and densely packed with mitochondria and contractile structures (Schaper et al., 1985; Barth et al., 1992). Both, their overall cell size, and intracellular structures constitute a significant hurdle for oxygen distribution. Moreover, oxygen must pass through different layers of actively respiring mitochondria to reach the innermost contractile compartments to satisfy their need for cellular respiration. It stands to reason that adequate oxygen distribution is additionally compromised under conditions of increased workload such as pressure overload and hypertrophy (Seiden et al., 1988; Sabbah, 2017), potentially rendering oxygen delivery a limiting determinant of ETS-driven redox responses. This would inevitably affect a number of processes intrinsically linked to ATP production including cellular redox balance, mitochondrial heat production and would eventually impair organ homeostasis and function (Suomalainen and Battersby, 2017; Bertero and Maack, 2018) (Figures 1B–D).

## 2 The emergence of oxygen gradients within cardiomyocytes

Given their large size, complex intracellular structures, and high metabolic activity, radial gradients of substrates, waste products, and ions conceivably occur within cardiomyocytes. The visualization of pronounced radial oxygen gradients in cultured rat cardiomyocytes was nevertheless a remarkable observation (Takahashi and Doi, 1996; Takahashi et al., 1998; Takahashi et al., 1999; Takahashi and Asano, 2002). Oxygen gradients emerged under conditions when cardiomyocytes were paced or treated with respiratory uncouplers. Although both interventions may appear as nonphysiological forms of stress, the observed oxygen gradients are likely to occur also *in vivo* in particular when oxygen becomes scarce or diffusion distances increase (Seiden et al., 1988; Sabbah, 2017). A major limitation to date in validating the biological significance of such gradients *in vivo* is owed to a lack of suitable oxygen biosensors and imaging systems. Yet, the possibility that oxygen may be unevenly distributed within cardiomyocytes raises several vital questions such as the nature of subcellular OXPHOS control mechanisms, and how organ homeostasis and function can be maintained despite presumably diverging metabolic signals. Interestingly, both uncoupling and pacing caused similar steep radial oxygen gradients. However, the corresponding NADH fluorescence showed divergent signals (Takahashi and Asano, 2002). In the case of uncoupling (maximal rate of respiration without ATP production), there was an increase in the NADH signal, indicative of hypoxia-induced respiratory inhibition. In the case of pacing, however, the NADH signal did not increase, which was interpreted as the presence of mechanisms that allow mitochondria with intact respiratory control to maintain electron transfer through the ETS even when oxygen becomes scarce (Takahashi and Asano, 2002). The nature of subcellular control mechanism remained elusive but several modes of spatial adaptation of bioenergetics to oxygen availability are conceivable, with each of which having a different effect on cardiac function.

## 3 Potential modes of spatial OXPHOS adjustment

### 3.1 Radial adjustment of OXPHOS activity may follow oxygen availability

Oxygen has long been proposed as the dominant regulator of both ATP-producing and ATP-consuming processes (Hochachka, 2003). Such regulatory control, however, implies that mitochondria are able to monitor the amount of available oxygen in order to adjust their energy metabolism accordingly. Indeed, recent evidence suggests that a key determinant of oxygen sensing may lie within the mitochondrial ETS itself (Sommer et al., 2017; Moreno-Domínguez et al., 2020; Sommer et al., 2020). Using such a mechanism, mitochondria may decrease the activity and adapt the composition of the OXPHOS system when oxygen levels are low. Conversely, OXPHOS activity could be gradually ramped up and reorganized during reoxygenation using the same mechanism (Figure 1B). In metabolically active cells of the size of cardiomyocytes with intracellular radial oxygen gradients (Takahashi and Doi, 1996; Takahashi et al., 1998; Takahashi et al., 1999; Takahashi and Asano, 2002), however, this would result in subcellular compartments being differentially energized, provided ATP content strictly follows oxygen availability. This type of spatial adaptation of bioenergetics harbors the risk of creating intracellular zones with different contractility, which may eventually lead to organ dysfunction.

Apart from limiting OXPHOS activity, a drop of oxygen below a critical threshold stabilizes hypoxia inducible factors (HIF), in particular HIF1α, thereby activating signaling cascades that rule cellular metabolism, mitochondrial ATP production, and overall signal transduction in a retrograde manner (Huang et al., 2004; Löfstedt et al., 2007; Krishnan et al., 2009; Liang et al., 2023; Sato and Takeda, 2023). In addition, the heart continuously adapts its metabolism dynamically, for example, through the glucose-fatty acid cycle or Randle cycle, where a decrease in the rate of fatty acid-β oxidation leads to a corresponding increase in the rate of glucose oxidation (Randle, 1998; Hue and Taegtmeyer, 2009). This is advantageous, *e.g.*, in the ischemic heart, since glucose oxidation produces more ATP per oxygen molecule consumed thus increasing cardiac efficiency under conditions of oxygen deprivation. However, if such shift in substrate usage occurs differentially in metabolically divergent zones within 1 cell and how this may be orchestrated remains elusive.

Hypoxic signaling could be avoided if oxygen levels are kept above the threshold critical for HIF1α stabilization (Hagen et al., 2003; Sato and Takeda, 2023). The observed stable NADH signal in paced cardiomyocytes (Takahashi and Asano, 2002) may equally be interpreted by a decrease of mitochondrial respiration in an attempt to maintain oxygen levels, provided the Krebs cycle activity closely follows ETS activity and therefore regenerates less NADH. The apparently stable NADH levels may thus indicate a lower turnover. It would be interesting to know whether local lactate levels increased as a result of compensatory stimulation of glycolysis during pacing and/or uncoupling. In addition to an oxygen sensor within the ETS (Sommer et al., 2017; Moreno-Domínguez et al., 2020; Sommer et al., 2020), extramitochondrial cues could prompt mitochondria to cease respiration, *e.g.*, when oxygen levels fall in relation to endogenously produced respiratory inhibitors such as nitric oxide. Disruption of mitochondrial respiration as a means of redistributing oxygen to non-respiratory oxygen-dependent targets including prolyl hydroxylases has previously been suggested (Hagen et al., 2003). In any case, a gradual adjustment of ETS-mediated redox responses likely leads to unevenly distributed ATP levels causing depolarization deficits and asynchronous contractile forces.

### 3.2 Proton-conducting mitochondrial fibers may render oxygen gradients irrelevant

The dilemma of unevenly distributed ATP levels due to potentially hypoxic regions was seemingly solved by an ingenious concept. It stressed the fact that striated muscles, skeletal muscle and heart, contain at least two spatially distinct mitochondrial subpopulations, *i.e.*, subsarcolemmal (SSM) and interfibrillar mitochondria (IFM) (Palmer et al., 1977). IFM located deep between the contractile structures should experience lower oxygen availability compared to SSM underneath the sarcolemma. Since mitochondria dynamically undergo fusion and fission, it was proposed that well-oxygenated SSM may support ATP production in supposedly less oxygenated IFM by forming proton-conducting fibers between the two compartments (Skulachev, 2001). Indeed, the existence of mitochondrial networks has been demonstrated in different cell types including fibroblasts and cardiomyocytes, in the latter called *streptio mitochondriale* (Amchenkova et al., 1988; Skulachev, 2001). In principle, the formation of *streptio mitochondriale* would allow ATP production in IFM even in the absence of local oxygen (Figure 1C). This elegant concept, however, poses a metabolic problem as the activity of upstream metabolic circuits such as the Krebs cycle are mandatorily dependent on redox reactions executed by the ETS. The Krebs cycle certainly shows tremendous plasticity in adapting to different metabolic conditions and may, for example, during hypoxia, operate in opposite directions (Chinopoulos, 2013). Indeed, an unusual accumulation of the Krebs cycle intermediate succinate was observed during cardiac ischemia (Chouchani et al., 2014; Chouchani et al., 2016), which may be beneficial during ischemia itself as it is thought to improve ischemic bioenergetics (Zhang et al., 2018). However, succinate accumulated during ischemia is rapidly oxidized upon reoxygenation, partially by reverse electron transport (RET), which produces excessive amounts of reactive oxygen species (ROS), a critical determinant of reperfusion injury (Chouchani et al., 2014; Chouchani et al., 2016). Fluctuating oxygen levels in cardiomyocytes could therefore lead to repetitive ROS-mediated injuries and altered Krebs cycle activities and/or accumulation of its intermediates. A pathologic accumulation of Krebs cycle intermediates, in turn, has consequences beyond bioenergetics as such metabolites fuel different stress response pathways, alter the metabolic flux and cause epigenetic alterations within the host cell and neighboring cells (Tretter et al., 2016; Chinopoulos, 2019; Martínez-Reyes and Chandel, 2020). Taken together, ATP level may be maintained in hypoxic cell segments by proton-conducting mitochondrial fibers, but it seems questionable how local restoration of ATP levels alone can exert beneficial effects if a disrupted ETS simultaneously causes a greater metabolic crisis.

### 3.3 Potential segmental adaption by mitochondrial hibernation

Equally possible is a radical third mode of adaptation, namely, the spatial downregulation of under-oxygenated mitochondria to complete inactivity, in particular the IFM compartment (Figure 1D). While a decrease in ETS function following radial oxygen gradients is readily conceivable, the obligatory dependence of the heart on oxidative metabolism appears to impose the maintenance of ATP-synthesizing activity by all means. The latter has led to the concept of proton conduction to maintain ATP synthesis in the absence of oxygen. Quite to the contrary, however, mitochondrial ATP synthase can dynamically shift between two opposing activity states: an ATP-synthesizing and an ATP-hydrolyzing state (Acin-Perez et al., 2023). The transition from ATP-synthesizing to ATP-hydrolyzing is triggered by a loss of membrane potential and thus correlates reciprocally with ETS function and presumably oxygen availability. At the molecular level, this switch to ATP hydrolysis is achieved by reversing the direction of the enzymatic reaction, whereby the ATP synthase mutates into an ATPase that pumps protons against a chemical gradient. The reversal of activity has long been recognized as a protective response to acute stress, such as myocardial infarction and oxygen starvation (St-Pierre et al., 2000). Surprisingly, it was recently described that the level of membrane potential may be heterogeneous along the inner membrane (Wolf et al., 2019). From this it follows that sections with high and low potential can alternate suggesting that ATP-synthesizing and ATP-hydrolyzing enzymes may coexist even within one mitochondrion. Therefore, a locally restricted shift in hypoxic cell segments towards ATP-hydrolyzing activity is possible. Of note, long-term activation of ATP hydrolysis in greater cellular regions, *e.g.*, the IFM compartment, inevitably would lead to ATP depletion. In support of this notion, overexpression of a mitochondrial protein that inhibits ATP hydrolysis (ATPase inhibiting factor 1, ATPIF1) and/or pharmacological inhibition of ATP hydrolysis conferred cardioprotection (Rouslin and Broge, 1989; 1993a; 1993b; Rouslin et al., 1995) and improved the phenotype of a mouse model of Duchenne muscular dystrophy (Acin-Perez et al., 2023). Caution is warranted, however, as the molecular mechanisms and biological effects of ATPIF1 expression appear to be multifaceted. While ATPIF1 may thus control a local reserve of ATP synthase that can be switched on or off upon demand (Romero-Carramiñana et al., 2023), it can also promote pathological cardiac remodeling and mitochondrial dysfunction when overexpressed (Pavez-Giani et al., 2021).

The inhibition of ATP-hydrolyzing activity of the mitochondrial ATP synthase emerged as particularly beneficial for the human heart. Unlike in rodents (fast heart rate species), in which ATP hydrolysis appears to be the predominant reaction when respiration is disrupted, the human heart mitochondrial ATP synthase (slow heart rate species) becomes catalytically inactive, presumably to preserve ATP levels (Rouslin and Broge, 1989; Rouslin and Broge, 1993a; Rouslin and Broge, 1993b; Rouslin et al., 1995). Together with impaired ETS-linked redox reactions, however, such inhibition of ATP hydrolysis (and proton translocation) leads to a decrease in mitochondrial membrane potential and makes mitochondria susceptible to degradation. More importantly, it impairs mitochondrial functions beyond ATP production, including ion homeostasis and protein import inducing a state of mitochondrial hibernation (metabolic quiescence) (Figure 1D). Mitochondrial hibernation may therefore also explain another unusual phenomenon, the metabolic stability of the mammalian heart. The canine heart, for instance, shows remarkably stable levels of energy metabolites despite large fluctuations in workload and oxygen consumption (Balaban et al., 1986), a phenomenon coined the “stability paradox” (Hochachka, 1999; Hochachka, 2003). The actual physiological role of *streptio mitochondriale* might thus be the delineation of metabolic segments and adaptation of contractile function as contractility in metabolically quiescent segments of cardiomyocytes is not conceivable. Taken together, an oxygen-controlled switch between activity and hibernation of mitochondria may allow the heart to adapt to different demands.

### 3.4 Could impaired substrate supply rule spatial OXPHOS adjustment?

To always maintain their contractile function, cardiomyocytes are metabolic omnivores (Aubert et al., 2013), they can flexibly switch between different substrates, and mitochondria are essential players in the maintenance of this flexibility (Muoio, 2014). Yet, local starvation of substrates may account for spatial adjustment of bioenergetics. Under physiologic conditions, fatty acid oxidation generates 60%–90% of ATP whilst oxidation of carbohydrates, and to a lesser degree ketones and amino acids provide the remaining 10%–40% (Bertero and Maack, 2018; Popoiu et al., 2023). The Randle cycle (Randle et al., 1963; Hue and Taegtmeyer, 2009) may thus become metabolically relevant when fatty acid oxidation outpaces intracellular transport mechanisms. Previous work, however, makes the concept of a spatial fatty acid deficiency seem unlikely. It has been demonstrated that the cytoplasmic transporter protein, fatty acid-binding protein (FABP), is present in abundance throughout cardiomyocytes suggesting that local fatty acid supply is not a rate-limiting condition (Luiken et al., 2003). Conversely, it was previously shown that high glucose levels can impair the activity of ETS complex I (Cannino et al., 2012). It is unclear, however, if a shift in the glucose to fatty acid ratio has the same effect. The metabolic flexibility may also force the heart to replace fatty acids by other substrates, *e.g.*, ketones (Ho et al., 2019; Ho et al., 2021). Ketone metabolism generally follows its availability and is thus independent from the mechanisms ruling the substrate switch described by the Randle cycle. However, ketones render the heart energetically less efficient (Ho et al., 2019; Ho et al., 2021) arguing against spatial substrate starvation as OXPHOS control mechanism but instead suggesting that a loss of spatial bioenergetic efficiency causes the heart to gradually become energy depleted.

Support for the idea of spatial energy starvation also comes from another observation. In pressure-overloaded human hearts, a significant correlation was observed between a decrease in ejection fraction and myocyte degeneration concomitant with an increase in autophagy (Hein et al., 2003). Mitochondrial hibernation (Figure 1D) decreases the membrane potential, which eventually predisposes mitochondria to degradation explaining why autophagy and OXPHOS activity are reciprocally regulated (Gustafsson and Gottlieb, 2009; Gottlieb and Mentzer, 2010; Rambold and Lippincott-Schwartz, 2011). Induction of mitochondrial hibernation due to insufficient oxygen availability may thus be the first step in a series of events that trigger autophagy and loss of cellular structures including mitochondria and myofibrils. Initially, this adaptive mechanism should lead to a decrease in contractility but eventually push the heart toward a tipping point at which the transition from compensated hypertrophy to contractile failure occurs. In support of this notion, we observed in a mouse model of inflammatory cardiomyopathy that enhanced tissue oxygenation alone was sufficient to restore cardiac contractility and reverse spatial dedifferentiation (Dhandapani et al., 2019). Furthermore, treatment with hyperbaric oxygen has previously shown beneficial effects, *e.g.*, it conferred cardioprotective effects under conditions such as carbon monoxide poisoning and ischemia (Swift et al., 1992; Sterling et al., 1993; Lund et al., 2000; Prockop and Chichkova, 2007; Jørgensen et al., 2013). Taken together, this suggests that oxygen is an important determinant of contractile adaptation and the development of contractile failure possibly through its spatial effect on bioenergetics.

## 4 Conclusion

Oxygen abundance is a prerequisite for mitochondrial bioenergetics and thus for cardiac contractile function. Therefore, oxygen sensing is vital for metabolic adaptation and is achieved by various means such as oxygen sensing within the ETS (Sommer et al., 2017; 2020; Moreno-Domínguez et al., 2020), reversal in the mode of direction of the ATP synthase or inhibition of its hydrolytic activity (Rouslin and Broge, 1989; Rouslin and Broge, 1993a; Rouslin and Broge, 1993b; Rouslin et al., 1995; Acin-Perez et al., 2023) and altered substrate selection (Randle, 1998; Hue and Taegtmeyer, 2009). Many of these processes are controlled by HIF proteins, which are stabilized or degraded in an oxygen-sensitive manner (Huang et al., 2004; Löfstedt et al., 2007; Krishnan et al., 2009; Liang et al., 2023; Sato and Takeda, 2023). Previously, critical attention has been paid to actual oxygen contents in tissues (Carreau et al., 2011) and the pitfalls arising from cell culture experiments carried out at ambient air (Ast and Mootha, 2019; Keeley and Mann, 2019). Relatively little is known about consequences of radial oxygen gradients that can build up within cells. A better understanding of this phenomenon, however, is important as it implies that different metabolic segments coexist within 1 cell.

In cultured rat cardiomyocytes, for instance, the formation of radial oxygen gradients was visualized (Takahashi and Doi, 1996; Takahashi et al., 1998; Takahashi et al., 1999; Takahashi and Asano, 2002). Since cardiomyocytes rely on OXPHOS for energy metabolism, the heterogeneity of oxygen distribution predicts the subsequent formation of ATP gradients that may cause depolarization deficits and asynchronous contractility. To avoid ATP gradients, it has been hypothesized that proton conduction along mitochondrial fibers to oxygen-depleted compartments may drive local ATP synthase activity (Skulachev, 2001). This concept, however, suffers from another shortcoming, *i.e.*, a stalling of metabolic circuits such as the Krebs cycle upstream of non-respiring mitochondria. Such a metabolic disruption also inevitably leads to an aberrant accumulation of metabolic intermediates such as succinate, which has consequences beyond energy metabolism (Chouchani et al., 2014; Martínez-Reyes and Chandel, 2020; Murphy and Chouchani, 2022).

Considering the various complications arising from different metabolic zones within one cardiomyocyte, we propose that spatial OXPHOS adaptation to a state of hibernation-like inactivity is the most likely response to local oxygen deprivation. In such a state, both the ETS-linked redox reactions and mitochondrial ATP synthase activity must be downregulated, including the hydrolyzing state of ATP synthase, which is thought to limit ATP exhaustion when oxygen is scarce (Rouslin and Broge, 1989; Rouslin and Broge, 1993a; Rouslin and Broge, 1993b; Rouslin et al., 1995). ATP levels in the cell center may nevertheless drop to a level impairing cardiac contractility. A hibernating-like state also causes a loss of membrane potential, which renders mitochondria susceptible to degradation. It stands to reason that the loss of mitochondrial mass due to degradation beyond a critical threshold defines the transition from cardiac contractile adaptation to contractile dysfunction.

Taken together, spatial regulation of bioenergetics may be the key mechanism for contractile adaptation to fluctuating tissue oxygen levels. If oxygen deprivation persists, loss of mitochondrial membrane potential and subsequently activation of autophagic activity may shift the heart towards the development of contractile failure. This puts spatial regulation of bioenergetics at the center of contractile adaptation, with oxygen being its chief determinant.
